# Difficulty in Diagnosing Anti-neutrophil Cytoplasmic Antibody-Related Vasculitis With Interstitial Pneumonia and in Ascertaining the Cause of Associated Hematochezia: A Case Report

**DOI:** 10.7759/cureus.34091

**Published:** 2023-01-23

**Authors:** Ryuichi Ohta, Kotaro Murakami, Yudai Tanaka, Tsuyoshi Mishiro, Chiaki Sano

**Affiliations:** 1 Community Care, Unnan City Hospital, Unnan, JPN; 2 Postgraduate Clinical Training Center, Shimane University Hospital, Izumo, JPN; 3 Internal Medicine, Unnan City Hospital, Unnan, JPN; 4 Community Medicine Management, Shimane University Faculty of Medicine, Izumo, JPN

**Keywords:** anti-neutrophil cytoplasmic antibody (anca)-associated vasculitis (aav), cytomegalovirus (cmv), non-specific interstitial pneumonia, general medicine, rural hospital

## Abstract

The diagnosis of rheumatological diseases is challenging among older patients with multimorbidity. Rheumatological diseases in older patients show varied symptoms, such as fatigue, fever, and appetite loss. We encountered an older woman with anti-neutrophil cytoplasmic antibody (ANCA)-related vasculitis complicated by cytomegalovirus (CMV) infection. The case was further complicated by hematochezia and was eventually diagnosed as CMV infection with adverse reactions to medications. This case highlights the difficulty of diagnosing ANCA-related vasculitis and dealing with the complications arising due to the side effects of therapy.

## Introduction

Diagnosing rheumatological diseases is challenging among older patients with multimorbidity [1,2]. Family physicians see patients with various symptoms, some of whom may suffer from rheumatological diseases [3]. Rheumatological diseases in older patients show various non-specific and common symptoms, such as fatigue, fever, and appetite loss [1]. Although joint pain is common in rheumatoid arthritis, other rheumatological diseases such as autoimmune vasculitis can also accompany joint pain [4]. Therefore, precise follow-up of patients’ symptoms is critical for effective diagnosis in the biomedical context.

However, to treat rheumatic diseases, dialogue with patients, their families, and other professionals is necessary due to disease severity and chronic progression from a psychosocial point of view [5]. In this study, we encountered an older woman with anti-neutrophil cytoplasmic antibody (ANCA)-related vasculitis complicated by cytomegalovirus (CMV) infection. Despite constant discussions with the patient and her family, our treatment did not result in a cure. This article reflects on the case using a three-step diagnosis framework.

## Case presentation

A 78-year-old female presented to our hospital with the chief complaints of exacerbation due to dyspnea, mild fever, and multiple joint pain. She lived in a rural community by herself. She divorced 20 years ago and had two sisters who lived far from the community. She did not possess a car and used public transportation for commuting. Financially, she relied only on pensions. Her daughter corroborated that she was independent and did not rely on her or anyone else. Her dyspnea had started four years before the presentation. Her primary care physician diagnosed her with idiopathic interstitial pneumonia and treated her with prednisolone (5 mg/day). As her symptoms did not improve, she began at-home oxygen therapy. Her symptoms gradually worsened, and one week before admission, she could not lead her usual life by herself. The patient was then brought to our hospital in an ambulance.

The vital signs at the visit were as follows: blood pressure: 110/67 mmHg; pulse rate: 110 beats/minute; body temperature: 37.2°C; respiratory rate: 24 breaths/minute; and oxygen saturation: 92% on room air. The patient was responsive and alert to time, place, and person. On physical examination, her joint pain was distributed peripherally and accompanied by one hour of morning stiffness. On physical examination, lung auscultation showed late crackles bilaterally, and both hands had multiple findings of arthritis in the proximal interphalangeal and metacarpophalangeal joints. The patient did not have a sputum culture. Laboratory tests revealed a high titer of rheumatoid factor, KL-6, and negative anti-cyclic citrullinated peptide antibody (Table 1).

**Table 1:** Initial laboratory test data of the patient. Ig: immunoglobulin; C3: complement component 3; C4: component 4; KL-6: Krebs von den Lungen-6; CCP: cyclic citrullinated peptide

Marker	Value	Reference
White blood cell count	14.30	3.5–9.1 × 10^3^/μL
Neutrophil differential count	85.5%	44.0%–72.0%
Lymphocyte differential count	7.5%	18.0%–59.0%
Monocyte differential count	6.0%	0.0%–12.0%
Eosinophil differential count	0.7%	0.0%–10.0%
Basophil differential count	0.3%	0.0%–3.0%
Red blood cell count	2.65	3.76–5.50 × 10^6^/μL
Hemoglobin level	9.2	11.3–15.2 g/dL
Hematocrit volume	26.5%	33.4%–44.9%
Mean corpuscular volume	100.1	79.0–100.0 fl
Platelet count	59.4	13.0–36.9 × 10^4^/μL
Erythrocyte sedimentation rate	117	3–15 mm
Total protein level	6.0	6.5–8.3 g/dL
Albumin level	2.0	3.8–5.3 g/dL
Total bilirubin level	0.4	0.2–1.2 mg/dL
Aspartate aminotransferase level	44	8–38 IU/L
Alanine aminotransferase level	25	4–43 IU/L
Alkaline phosphatase level	75	106–322 U/L
γ-Glutamyl transpeptidase level	171	<48 IU/L
Lactate dehydrogenase level	171	121–245 U/L
Blood urea nitrogen level	23.3	8–20 mg/dL
Creatinine level	0.78	0.40–1.10 mg/dL
Estimated glomerular filtration rate	54.0	>60.0 mL/min/L
Serum sodium level	136	135–150 mEq/L
Serum potassium level	4.0	3.5–5.3 mEq/L
Serum chloride level	101	98–110 mEq/L
Creatine kinase level	73	56–244 U/L
C-reactive protein level	15.12	<0.30 mg/dL
IgG level	1624	870–1,700 mg/dL
Antinuclear antibody level	<40	<40
C3 level	161	86–160 mg/dL
C4 level	32	17–45 mg/mL
KL-6 level	421	105.3–401.2 U/mL
Rheumatoid factor level	103	<15 IU/mL
CCP antibody level	<0.6	<5 U/mL

**Figure 1:**
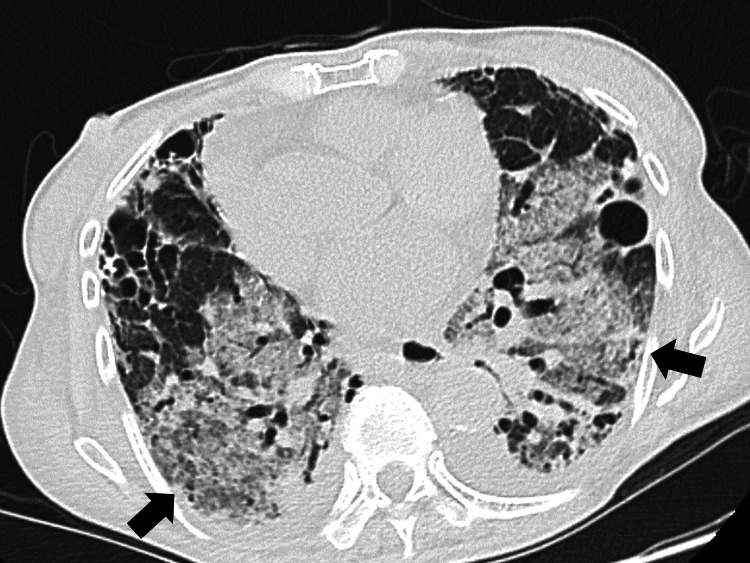
Computed tomography image showing interstitial infiltration in bilateral lungs.

Chest radiography and computed tomography showed interstitial infiltration in both lungs (Figure 1).

Hand and foot X-rays did not reveal any bone deformities or calcification. Blood cultures for bacteria were negative. We diagnosed her with exacerbation of interstitial pneumonia with rheumatoid arthritis and treated her with prednisolone 30 mg/day. Ten days after the initiation of the treatment, her dyspnea continued and exacerbated with deoxygenation leading to a SpO_2_ of 90% (oxygen treatment of 6 L).

On the 11th day of admission, we involved the patient and her family in the decision-making process and discussed the future course of treatment. Despite the risks, the patient wished to continue treatment. Still, one of her daughters was averse to the risk of tracheostomy in the intensive care setting and wanted to discuss the possibility of palliative care with the patient. The patient, however, strongly advocated for intensive care, and the daughter finally agreed with the patient’s decision. On the 13th day of admission, intratracheal intubation and artificial ventilation were performed. The drug dosage was increased to 1,000 mg/day of methylprednisolone for three days and 500 mg/day of cyclophosphamide. An additional laboratory test revealed a high titer of 126 U/mL for P-ANCA. Consequently, the patient was further diagnosed with ANCA-related vasculitis and interstitial pneumonia. On the 17th day of admission, her symptoms progressed without other signs of infection, including pneumocystis pneumonia. Rituximab (500 mg) was also administered for the remission of vasculitis.

**Figure 2:**
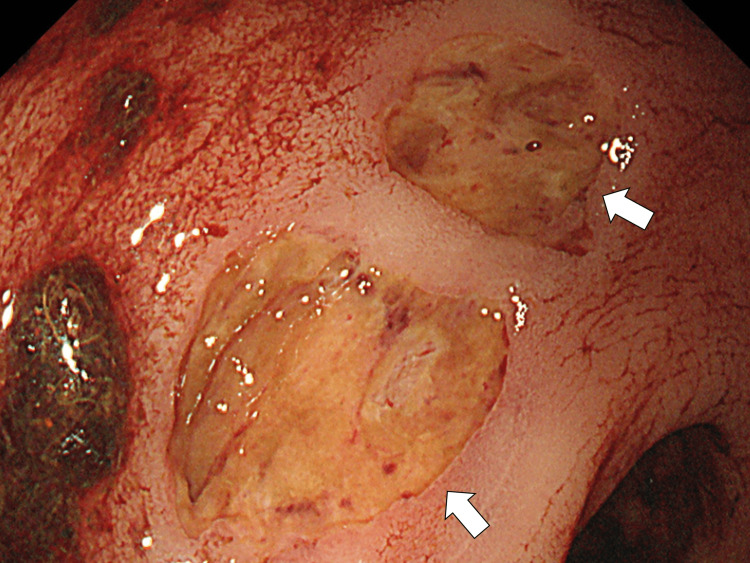
Colonoscopy revealing multiple ulcers in the sigmoid colon.

Her respiratory condition gradually improved, but hematochezia was observed one week after rituximab administration. First, we suspected this to be a complication of rituximab administration and closely monitored it. A week later, hematochezia recurred, and her respiratory condition worsened. Lower endoscopy showed multiple ulcers in the rectum and the sigmoid colon, showing infection with CMV, as confirmed by CMV antigenemia (Figure 2).

In response, the treatment with 500 mg/day of ganciclovir was initiated. The hematochezia was cured, but her respiratory condition did not improve. The patient’s condition was reassessed and discussed with her daughter to decide on further treatment. She decided on hospice care; therefore, we had the consent not to perform cardiopulmonary resuscitation when the patient was arrested. As her respiratory condition progressively deteriorated, she died due to respiratory failure on the 36th day of admission.

## Discussion

This case shows the difficulty of diagnosing ANCA-related vasculitis that causes interstitial pneumonia and ascertaining the cause of hematochezia ranging from the side effect of the administered drug (rituximab) to CMV infections. In the decision-making process for treatment, the conflict and dialogue between the patients and their families should be respected and mediated by family medicine doctors. We discuss and reflect on the three-stage diagnosis suggested by MacWhinney [6]. The framework of the three-stage diagnosis consists of clinical diagnosis, personal perception of the disease, and ecological diagnosis, which can be useful for comprehensively assessing patients’ conditions.

Clinical diagnosis

The patient was clinically diagnosed with ANCA-related vasculitis and interstitial pneumonia complicated by CMV infection. The diagnostic process was complicated because of the varied symptoms. Chronic joint pain requires consideration of various differential diagnoses, including rheumatoid arthritis and other rheumatological diseases [7]. Among older patients, ANCA-related vasculitis has a relatively higher prevalence than in younger patients [4]. Various rheumatic diseases can induce interstitial pneumonia. Common rheumatological diseases include rheumatoid arthritis, systemic sclerosis, and dermatomyositis [8]. In addition, ANCA-related vasculitis can also cause interstitial pneumonia, typically in older patients [4]. For an effective diagnosis of ANCA-related vasculitis, older patients with joint pain and interstitial pneumonia are generally considered [4]. In this case, the initial diagnosis was rheumatoid arthritis with interstitial pneumonia based on the prevalence of rheumatoid arthritis among the older population. However, concerning the timing of exacerbation, the measurement of ANCA was ordered and was positive. Based on this diagnosis, we recommend assessing ANCA in older patients with interstitial pneumonia.

Rituximab requires consideration of side effects and infections when patients experience hematochezia. In this case, one consideration regarding hematochezia was the side effects of rituximab on the intestinal wall [9]. The side effects may be withdrawn by discontinuing the medication. Another consideration is opportunistic infections involving the colon, particularly caused by fungi and CMV [9]. In this case, antigenemia for CMV was positive, suggesting a CMV infection. However, opportunistic infections usually occur several weeks after starting immunosuppressant therapy. Reflecting on the clinical course, although it was a rare presentation, the first episode of hematochezia could be considered a CMV infection. In treatment with rituximab, opportunistic infections should be considered during the initial phase of treatment.

Personal diagnosis

Treatment decisions should include patient preferences and ecological aspects [6]. Regarding personal preference, the patient hoped to live and continue treatment, even in a critical situation. I wanted to respect her decision. Her decision could have been affected by her life. She lived alone and wanted to control and prepare for the end of life. The motivation for her life could have driven her to receive intensive care. Her daughter could hear these personal perspectives.

Discussions involving families should also be facilitated for effective and shared decision-making [6]. In this case, the patient’s daughter initially disagreed with the patient’s desire for intensive care. However, through robust discussions between the patient and the daughter, they agreed on intensive care. Shared decision-making is essential for intensive treatment, which facilitates appropriate treatment for patients taking their circumstances into account. Family physicians should, therefore, be familiar with shared decision-making to make clinical decisions.

Ecological diagnosis

Ecological diagnoses include patients’ living places, primary care doctors, and family relationships. This patient visited a rural primary care doctor regularly. The rural clinic physician suggested that the patient visit general hospitals to investigate her symptom exacerbation further. However, the patient was reluctant to visit the hospital because of its accessibility [10]. As previous research has suggested, rural settings that lack adequate healthcare resources could lead to adverse health outcomes [11]. In addition, living alone may have affected her help-seeking behavior (HSB) [12]. She did not depend on her daughters, so she might not have been in contact with them. As her financial condition was unknown because of limited care duration, in rural contexts, socioeconomic issues regarding the fare of public transportation and time taken to reach hospitals might have affected her HSB. In rural contexts, modifying HSB according to the patient’s lifestyle and culture should be discussed further [13,14].

## Conclusions

This diagnostic error led to a loss of confidence and a sense of regret concerning poor logical thinking in making the diagnosis. To avoid these mistakes, various patterns of ANCA-related vasculitis mimicking rheumatoid arthritis were revisited. In addition, it was realized that time pressure and mental stress could impinge logical thinking. Regardless of the time pressure, additional tests should always be performed to avoid misdiagnosing ANCA vasculitis among older patients because ANCA-related vasculitis is relatively common. In treating critical diseases, shared decision-making can improve the understanding of patients and families regarding treatments, and the process can establish a mutually respectful relationship among medical professionals and families.
